# Identification of XylR, the Activator of Arabinose/Xylose Inducible Regulon in *Sulfolobus acidocaldarius* and Its Application for Homologous Protein Expression

**DOI:** 10.3389/fmicb.2020.01066

**Published:** 2020-05-26

**Authors:** Nienke van der Kolk, Alexander Wagner, Michaela Wagner, Bianca Waßmer, Bettina Siebers, Sonja-Verena Albers

**Affiliations:** ^1^Institute of Biology II, Molecular Biology of Archaea, University of Freiburg, Freiburg, Germany; ^2^Molecular Enzyme Technology and Biochemistry, Environmental Microbiology and Biotechnology, Centre for Water and Environmental Research, University of Duisburg-Essen, Essen, Germany; ^3^Biotechnologie, Hochschule Niederrhein, Krefeld, Germany

**Keywords:** genetics, genetic tools, transcriptional regulator, xylose metabolism, archaea, *Sulfolobus*

## Abstract

The thermophilic archaeon *Sulfolobus acidocaldarius* can use different carbon sources for growth, including the pentoses D-xylose and L-arabinose. In this study, we identified the activator XylR (*saci_2116*) responsible for the transcriptional regulation of the pentose transporter and pentose metabolizing genes in *S. acidocaldarius*. A *xylR* deletion mutant showed growth retardation on D-xylose/L-arabinose containing media and the lack of transcription of the respective ABC transporter. In contrast to so far used promoters for expression in *S. acidocaldarius*, the xylR responsive promoters have a very low background activity. Finally, two XylR dependent promoters next to the long-established maltose inducible promotor were used to construct a high-throughput expression vector system for *S. acidocaldarius* to efficiently clone and express proteins in *S. acidocaldarius*.

## Introduction

Although metabolically less versatile than *Saccharolobus solfataricus*, the thermophilic archaeon *S. acidocaldarius* grows on different sugar substrates, including dextrin, sucrose, D-glucose, D-xylose, and L-arabinose (Grogan, 1989; Hatzinikolaou et al., 2001). Recently, the transporter responsible for pentose uptake in *S. acidocaldarius* was identified: XylF (*saci_2122*), XylG (*saci_2121*), XylH (*saci_2120*) (Wagner et al., 2018). Also, the pentose metabolizing genes involved in the aldolase independent Weimberg pathway were characterized. The promiscuous glucose dehydrogenase 1 (GDH1, Saci_1079), D-xylonate/L-arabinoate dehydratase (D-KDXD/L-KDAD, Saci_1039), the 2-keto-deoxy-D-xylonate/L-arabinoate dehydratase (D-KDXD/L-KDAD, Saci_1939), and the α-ketoglutarate semialdehyde dehydrogenase (α-KGSADH, Saci_1938) degrade the metabolites to α-ketoglutarate, a central intermediate of the citric acid cycle. Our studies demonstrated that *S. acidocaldarius* strain MW001 utilizes only the Weimberg pathway and not the aldolase-dependent Dahms pathway (Wagner et al., 2018). Previous ^13^C labeling studies in *S. acidocaldarius* (DSM 639) grown on D-xylose revealed that both routes, the Weimberg and Dahms pathway, are operative *in vivo* at similar ratios (Nunn et al., 2010).

Like most characterized sugar transporters in Archaea, the pentose transporter in *S. acidocaldarius* belongs to the ATP-binding cassette (ABC) transporter systems. Whereas the other characterized archaeal sugar ABC transporters fall into the carbohydrate uptake transporters 1 (CUT1) family, this pentose transporter belongs to the carbohydrate uptake transporter 2 (CUT2) family and was obtained via horizontal gene transfer from bacteria (Wagner et al., 2018).

The pentose transporter operon encodes for a 10-transmembrane-spanning subunit and only a single nucleotide-binding domain, probably forming a homodimer (Wagner et al., 2018).

The promotor region of the genes encoding the *S. acidocaldarius* pentose transporter as well as pentose degrading enzymes contain a cis-regulatory element the so-called “Ara-box,” which is conserved among different *Sulfolobales* and has been studied intensively and been used in *Sulfolobus* expression systems (Brouns et al., 2006; Lubelska et al., 2006; Peng et al., 2009). The palindromic motif can be found in the promotor regions of genes upregulated in the presence of L-/D-arabinose or D-xylose (Brouns et al., 2006; Peng et al., 2009; Wagner et al., 2018). Notably, although *S. acidocaldarius* is in contrast to *Saccharolobus solfataricus* not able to utilize D-arabinose as carbon source, induction of the sugar-binding protein (SBP) promoter (*saci_2122*) was observed in the presence of D-arabinose (Brouns et al., 2006). Although the Ara-box has been known for a while, the respective Ara-box binding transcription factor (TF) has not been identified yet. In Archaea, multiple components of the RNA polymerase (RNAP) show homology to the eukaryotic RNAPII in both structure and organization (Zillig et al., 1979; Langer et al., 1995; Werner, 2007), and they are often considered as a simplified version of the eukaryotic machinery (Bell and Jackson, 2001). However, the eukaryal-like transcription machinery is regulated by transcription factors (TF) that resemble those in bacteria (Aravind et al., 2005; Ouhammouch et al., 2005). Like in bacteria, archaeal TFs are often homodimers recognizing specific semi-palindromic DNA motifs in the promotor region (Peeters et al., 2004, 2013; Keese et al., 2010). The largest fraction of TFs contain an HTH (helix-turn-helix) domain, although RHH (ribbon-helix-helix) motif and the zinc ribbon are not uncommon (Aravind and Koonin, 1999; Aravind et al., 2005).

Here, we present the identification of the transcriptional activator XylR (saci_2116), which is essential for the transcription of genes in the xylose/arabinose uptake and degradation pathway in *S. acidocaldarius*. Furthermore, we established a high-throughput expression system driven by carbon source induction. The new expression system provides functional expression plasmids based on the FX-cloning principle (Geertsma and Dutzler, 2011), which were optimized to facilitate high-throughput expression in the thermophile *S. acidocaldarius.* Like other expression systems in *Sulfolobales* (Albers et al., 2006; Peng et al., 2012), expression can be induced by addition of different carbon sources; two pentose (i.e., D-xylose, L-/D-arabinose)-inducible promotors (P_saci___1938_ and P_saci___2122_) and one with either maltose or dextrin induction (P_saci___1165_). In this new set of vectors genes can be cloned from an entry vector with a ligation independent method in one step in various different expression vectors. In addition, traditional restriction site dependent cloning can be performed. This will facilitate efficient screening for optimal expression conditions of homologous and recombinant proteins in *S. acidocaldarius*.

## Materials and Methods

### Strains and Growth Conditions

*Sulfolobus acidocaldarius* was grown aerobically in a 75°C incubator in basal Brock medium at pH 3.5 (Brock et al., 1972). The medium was supplemented with 0.1% w/v NZ-Amine and with different carbon sources e.g., 0.2% dextrin w/v, 0.2% D-arabinose w/v, 0.2% L-arabinose w/v, 0.2% D-xylose w/v, 0.2% sucrose w/v or 0.2% D-glucose w/v, unless specified otherwise. MW001 and other deletion mutants (Supplementary Table S1) were supplemented with 10 μg/ml uracil for growth. The growth was monitored by determination of the optical density at 600 nm. *Escherichia coli* Top10 and ER1821 (New England Biolabs, Frankfurt am Main, Germany), used for cloning and methylation of the plasmids, were grown in Lysogenic broth medium (10 g/L tryptone, 5 g/L yeast, 10 g/L NaCl) at 37°C supplemented with the respective antibiotics.

### Construction of Markerless Deletion of *saci_2116*

To obtain a markerless deletion of *saci_2116* plasmid pSVA595 was constructed. With a PCR the last ∼500 bp of *saci_2116* were amplified and cloned into the MCSII of pSVA431 using restriction enzymes *Kpn*I and *Nco*I. Approximately 500 bp region upstream and downstream of the target gene were PCR amplified. The two fragments were annealed by overlap extension PCR and cloned with *Pst*I and *Bam*HI into the MCSI site of the previously obtained plasmid. The two most outward primers (upstream and downstream) were used to create a linear fragment via PCR, which was used for transformation to competent MW001 cells as described in Wagner et al. (2012).

### Transformation of *S. acidocaldarius*

MW001 cells were made competent according to (Wagner et al., 2012). Methylated plasmids or linear DNA fragments were transformed by using a Gene Pulser Xcell (BioRad, München, Germany) at 2000 V, 600 Ω, 25 μF in 1 mm cuvettes. After recovery in Brock medium at 75°C, as described in Wagner et al. (2014), the cells were plated on gelrite plates lacking uracil but containing 0.2% Dextrin and 0.1% N-Z-Amine and grown for 5 days at 75°C in a plastic box to prevent the plates from drying-out. For in genome deletions the transformed cells were grown in liquid medium without uracil and plated on second selection gelrite plates containing 200 μg/ml 5-FOA (5-Fluoroorotic acid) and 10 μg/ml uracil.

### Growth Curve of MW001 and MW413

The transformed cells were grown in Brock with 0.2% NZ-Amine, to prevent pre-adaptation till an OD600 nm between 0.3 and 0.5. The next day 5 ml Brock with 0.1% NZ-Amine or 0.1% NZ-Amine supplemented with 0.2% L-arabinose, 0.2% D-xylose or 0.2% glucose were inoculated with cells to an OD600 nm of 0.01. The optical density was measured over a course of 72 h. All cultures were grown in biological triplicates and technical triplicates.

### Complementation Studies

For the complementation of the Δ*xylR* strain (MW413), pSVA-xylR was constructed by replacing the P_ara_ (P*_saci___2122_*) and the LacZ gene in pSVAaraFX-stop by *xylR*, including its own promoter. The fragment was amplified by PCR and cloned into the pSVAaraFX-stop with *Sac*II and *Sal*I, creating pSVA-xylR.

One of the proteins of *S. islandicus* (*m1627_2327*) showed homology with XylR (54% identity, 81% positives) and *in trans* complementation was attempted with this gene. *m1627_2327* was amplified by PCR. To make sure the start site was recognized by *S. acidocaldarius*, the *xylR* promoter (P_saci___2116_) was cloned upstream of *m1627_2327* by overlap PCR. The fragment was cloned into pSVAaraFX-stop with *Sac*II and *Xho*I, resulting in pSVA-S.isl-xylR. The Δ*xylR* strain (MW413) was transformed with pSVA-xylR, pSVA-S.isl-xylR, and pSVAaraFX-empty, the latter plasmid serving as negative control. pSVAaraFX-empty was created by replacing the lacZ gene by Stop codons in all three frames. Complementation was assessed by growth comparison in 5 ml Brock medium supplemented with 0.1% N-Z-Amine or 0.1% N-Z-Amine and 0.2% L-arabinose in glass tubes covered with lid.

### Quantitative RT-PCR

MW001 and MW413 were grown in triplicate in medium supplemented with 0.1% N-Z-Amine or 0.1% N-Z-Amine and 0.2% sugar (L-arabinose, D-arabinose, D-xylose, dextrin, sucrose or glucose). Cell cultures (14 ml) were harvested at mid-exponential phase (OD_600_ ∼ 0.4). RNA was isolated as described previously (Lassak et al., 2012). cDNA synthesis was carried out with QuantiNova^TM^ Reverse Transcription Kit (Qiagen, Venlo, Netherlands) according to the manufactures protocol. The quantitative RT-PCR was performed in the Magnetic Inducible Cycler (MIC) (Bio molecular systems, Upper Coomera, Australia) with 2xqPCRBIO SyGreen mix Lo-ROX (PCRBiosystems, London, United Kingdom) by following the manufacturer’s instruction. Probes were activated by heating to 95°C for 2 min, followed by 40 cycles of 95°C for 5 s and 60°C for 20 s. The transcription levels were analyzed with gene-specific primers (Supplementary Table S3) for the sugar binding protein (*saci_2122*). The cq-values were normalized to the cq-values of the housekeeping gene *secY* (*saci_0574*).

### Plasmid Construction and Sample Preparation ONPG Assay xylR

pSVAaraFX-lacS-xylR is based on the expression vector pSVAaraFX-Stop. This vector contains lacS, cloned into the expression cassette as reporter gene via FX cloning and additionally the *xylR* regulator gene under its own promoter, inserted in the backbone via restriction with *Pst*I, *Xma*I. In this vector two different point mutations in the Ara-box of the ara-promoter were generated via round PCR, resulting in pSVAaraBox1 and pSVAaraBox2. Prior to transformation into *S. acidocaldarius* plasmids were methylated in *E. coli* ER1821 to prevent restriction by the SuaI restriction enzyme.

The transformed cells were grown in Brock with 0.2% NZ-Amine, to prevent pre-adaptation. The next day 5 ml Brock with 0.1% NZ-Amine and 0.2% L-arabinose or 0.2% dextrin were inoculated from a pre-culture and grown till OD600 nm 0.3–0.4. Cell pellets were collected by spinning down 2 ml at 16,000 × g for 5 min. The pellets were stored at −20°C. The experiment was performed in triplicate.

### ONPG (β-Galactosidase) Assay

Cell pellets were lysed in Z-buffer (pH 7) (10 mM KCl, 1 mM MgSO_4_, 60 mM Na_2_HPO_4_ and 40 mM NaH_2_PO_4_) supplemented with 1% Triton-X to a theoretical OD of 3.2. The assay was performed at 56°C in a 96-well plate with 20 μl cell lysate, 170 μl Z-buffer and 10 μl ONPG (σ-nitrophenyl-β-d-galactopyranoside) solution (40 μM). The conversion rate of ortho-nitrophenol was measured at 410 m in a microplate reader (CLARIOstar, BMG Labtech, Ortenberg, Germany). Additionally, the protein concentration of the samples was measured with the BCA kit according to manufactures protocol (Serva Electrophoresis GmbH, Heidelberg, Germany).

The galactosidase activity was calculated in Miller Units with the equation:

β-g⁢a⁢l⁢a⁢c⁢t⁢o⁢s⁢i⁢d⁢a⁢s⁢e⁢a⁢c⁢t⁢i⁢v⁢i⁢t⁢y=60,000x(A410⁢n⁢m()t⁢2-t⁢1-autolysisat 410nm(t⁢2-t⁢1))Δ⁢t⁢i⁢m⁢e⁢x⁢v⁢o⁢l⁢u⁢m⁢e⁢o⁢f⁢s⁢a⁢m⁢p⁢l⁢e⁢[m⁢l]x⁢p⁢r⁢o⁢t⁢e⁢i⁢n⁢c⁢o⁢n⁢c⁢e⁢n⁢t⁢r⁢a⁢t⁢i⁢o⁢n⁢[m⁢gm⁢l]

### Construction of Expression Plasmids for *Sulfolobus acidocaldarius*

For a detailed description (see Supplementary Table S2). In brief, the pSVAmZ-SH10 plasmid was used as backbone for all expression plasmids (Wagner et al., 2014). Two silent single base pair mutations were introduced to remove the *Sap*I restriction sites from the backbone, which would spoil FX-cloning. In return the cloning cassette was modified and two *Sap*I restriction sites compatible to the FX-cloning system were integrated. PCR products of ∼100 bp base pairs upstream of *saci_2122* (pSVAaraFX-) or *saci_1938* (pSVAxylFX-) were generated to create the pentose inducible expression plasmids, and a PCR product of ∼150 bp base pairs upstream of *saci_1165* (pSVAmalFX-) for the maltose/dextrin inducible promoter. With an overlap PCR the N-terminal tags were fused to the different promoters and cloned into the backbone with *Sac*II and *Nco*I. The overlapping primers containing the C-terminal tags were annealed and cloned into the backbone with *Apa*I and *Xho*I.

### Promotor Activity Assay on Different Carbon Sources

To measure the promotor activity in presence of different sugars, a PCR fragment of *lacS* (*sso_3019*) was cloned into pSVAmalFX-Stop, pSVAxylFX-Stop, and pSVAaraFX-Stop according to the FX-method with *Sap*I, creating pSVAmalFX-lacS, pSVAxylFX-lacS, and pSVAaraFX-lacS respectively.

Transformed MW001 cells were grown on Brock with 0.1% NZ-Amine and 0.3% sucrose. The next day 5 ml Brock was inoculated with 0.1% NZ-Amine and 0.3% of the respective sugar: L-arabinose, dextrin, D-glucose, maltose, sucrose or D-xylose. After 18 h cell pellets were collected by spinning down 2 ml at full speed for 5 min in a table top centrifuge. The pellets were stored at −20°C until used for the ONPG assay.

## Results

### Identification of XylR

**FIGURE 1:**
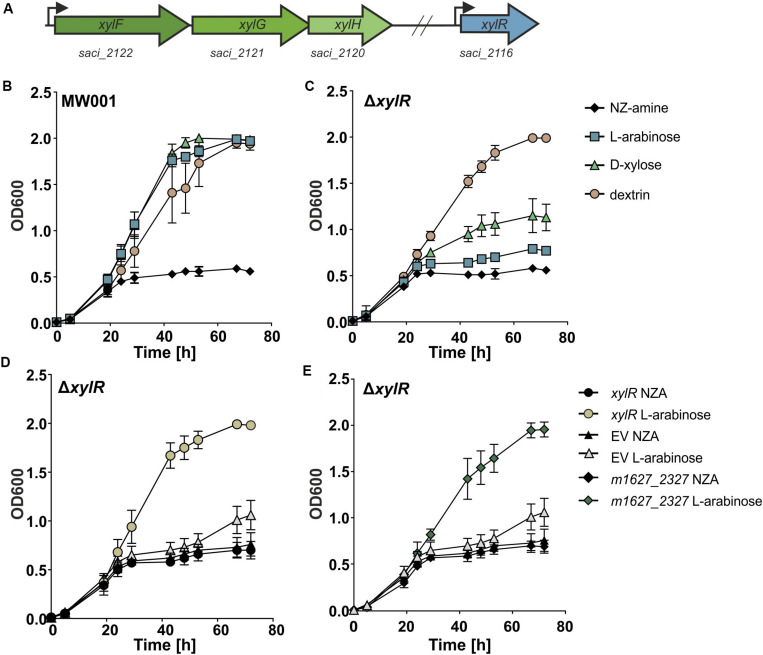
**(A)** A schematic representation of the genes encoding the pentose transporter (in green) and *xylR* (in blue) *S. acidocaldarius*. The transcription start sites are depicted with an arrow. **(B)** Growth comparison of parental (MW001) and **(C)** *xylR* (Δ*saci_2116*) deletion strain on 0.1% N-Z-Amine and 0.1% N-Z-Amine supplemented with 0.1% L-arabinose, 0.1% D-xylose or 0.1% glucose. Complementation of the Δ*xylR* strain with *S. acidocaldarius xylR* **(D)** and with the *S. islandicus* homolog of *xylR* (m1627_2327) **(E)**.

Recently, we identified the pentose transporter in *S. acidocaldarius* (Wagner et al., 2018). This ABC-transporter from the CUT-2 family is most homologous to bacterial transporters. Interestingly, the promotor region comprises a palindromic motif known as the Ara-box (Wagner et al., 2018). The Ara-box is conserved among different *Sulfolobales* species and shown to be essential for the D-arabinose inducible promoter activity (Supplementary Figure S1) (Brouns et al., 2006; Peng et al., 2009). However, the transcription factor regulating this promoter was still unidentified. Downstream of the *S. acidocaldarius* pentose transporter (*saci_2120*–*2122*), the gene *saci_2116* is located, which is a predicted transcriptional regulator containing a helix-turn-helix (HTH) domain (Figure 1A). This gene will now be referred to as *xylR*.

To elucidate the importance of XylR for the growth of *S. acidocaldarius* on pentoses, a markerless deletion of *xylR* was made in the uracil auxotroph parent strain MW001 (Wagner et al., 2012). The effect of the knock-out of *xylR* on the growth of *S. acidocaldarius* was tested by growth experiments on different carbon sources (Figures 1B,C). When grown on L-arabinose or D-xylose the *xylR* deletion mutant exhibited a strong growth reduction. However, the Δ*xylR* strain showed no phenotype when grown on NZ-Amine alone or NZ-Amine and dextrin, excluding a general growth defect of the mutant. Also, growth of the deletion mutant could be fully restored when complemented with a plasmid expressing *xylR* from its own promoter (pSVA-xylR) (Figure 1D). This showed that *xylR* was essential for growth of *S. acidocaldarius* on pentoses like L-arabinose and D-xylose.

As mentioned before, the Ara-box is conserved in the promoter region of a variety of genes among different *Sulfolobales* species and shown to be essential for their promoter activity (Peng et al., 2009). Therefore, it is very likely that the putative regulator(s) binding to this DNA motif should be conserved among the different *Sulfolobus* species. The protein sequence of XylR showed homology with predicted regulators in the genomes of *S. islandicus* and *S. solfataricus* P1 with around 50% identity. Therefore, *xylR* of *S. islandicus* (*m1627_2327*) was expressed in the *S. acidocaldarius* deletion strain Δ*xylR* to test whether it can complement and thereby fully restore growth of the Δ*xylR* deletion strain on pentoses. The promoter of *saci_2116* was cloned upstream of *m1627_2327* in a plasmid (pSVA-S.isl-xylR) and transformed into competent Δ*xylR S. acidocaldarius* cells. The transformed cells were grown in Brock medium supplemented with 0.1% N-Z-Amine or 0.1% N-Z-Amine and 0.2% L-arabinose. From the growth curve it was clear that while the cells complemented with the empty plasmid (pSVAaraFX-empty) showed no growth stimulation in media supplemented with L-arabinose, the cells expressing M1627_2327 grew to higher ODs (Figure 1E). These results suggest that M1627_2327 is the XylR homolog in *S. islandicus*.

### Regulation of the *xyl* Operon in the *XylR* Deletion Mutant

**FIGURE 2:**
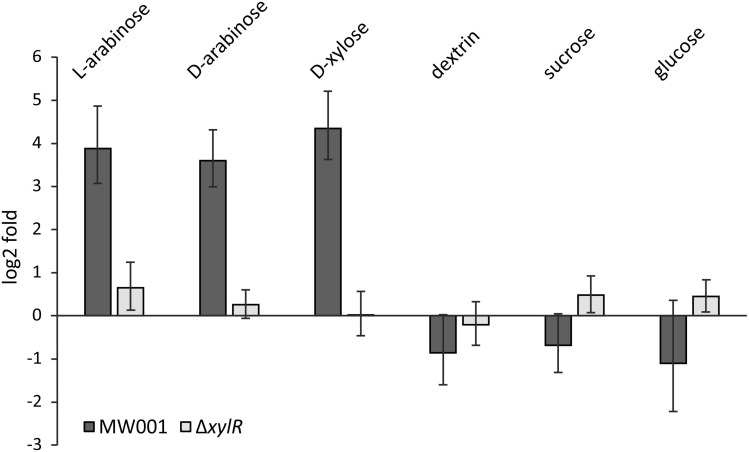
Transcription levels of *xylF* (*saci_2122*) in the parental strain MW001 and the Δ*xylR* deletion mutant when grown on different sugars. The strains were cultured on 0.1% N-Z-Amine supplemented with the different sugars (0.2% L-arabinose, 0.2% D-arabinose, 0.2% D-xylose, 0.2% dextrin, 0.2% sucrose or 0.2% D-glucose). Bars indicate the sugar-specific transcription compared to cells only grown on N-Z-Amine on log_2_-fold.

To confirm the role of XylR as an activator of the xyl/ara promoter, transcription levels of D-xylose/D-/L-arabinose inducible genes were tested in MW001 and the *xylR* deletion strain. Expression levels of the sugar binding protein of the pentose importer – XylF – (*saci_2122*) were tested in cells grown on different carbon sources for both MW001 and Δ*xylR* (Figure 2). In the parental strain, *xylF* transcription is highly upregulated in the presence of pentoses (i.e., L-arabinose, D-xylose, and also D-arabinose, where the latter does not serve as carbon source for *S. acidocaldarius*) but not in the presence of hexoses. However, in the absence of *xylR* no significant induction can be detected in any condition. The same results were obtained when the expression levels of the KDXD/KDAD encoding gene (*saci_1939*), essential for the degradation of pentoses, were tested (Supplementary Figure S2).

### XylR Dependent Promotor Activity Assay

To verify that XylR indeed binds to the pentose inducible promoters, DNA binding assays with purified protein would be the method of choice. However, any attempt to express XylR in *E. coli* or in *S. acidocaldarius* failed as the addition of an affinity tag seemed to destabilize the protein.

**FIGURE 3:**
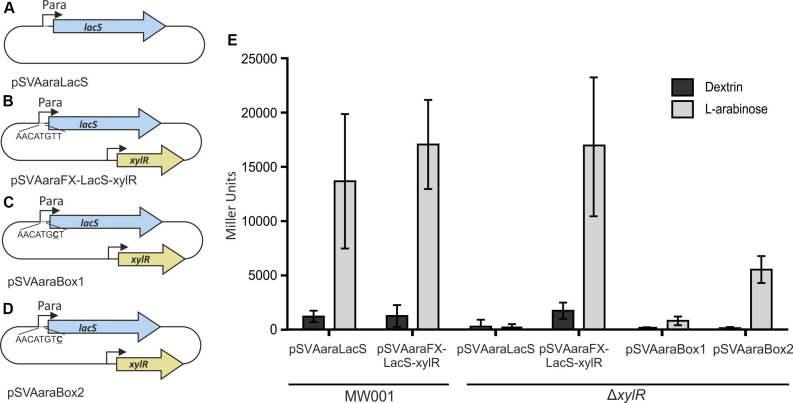
Schematic illustration of the different vector constructs **(A–D)**. pSVAaraLacS **(A)** and pSVAaraFX-LacS-xylR **(B)** expressing *lacS* under control of the ara-promotor (P*saci_2122*) and the Ara-box mutants pSVAaraBox1 **(C)** and pSVAaraBox2 **(D)** are depicted. pSVAaraFX-LacS-xylR **(B)** contains in addition *xylR* under control of its own promotor. The respective mutation in the Ara-box **(C,D)** is underlined. **(E)** β-galactosidase activity of MW001 and Δ*xylR* transformed with the different vector constructs. The strains were cultured in 0.1% N-Z-Amine and supplemented with either 0.2% L-arabinose or 0.2% dextrin. The β-galactosidase activity **(E)** is shown in Miller Units.

Therefore, we used an ONPG assay, to verify that XylR is responsible for the induction of the pentose promoter. *LacS* was expressed in MW001 and MW413 (Δ*xylR)* from pSVAaraLacS (without *xylR*) or pSVAaraFX-LacS-xylR (with *xylR*). The cell extracts of the transformants grown on L-arabinose or dextrin were tested for hydrolysis of ONPG (Figure 3). When *xylR* was present, either in the genome or on the plasmid, the promotor shows high activity in the presence of L-arabinose and limited activity when the cells were grown on dextrin. However, activity is completely abolished in the absence of *xylR*. To confirm the basic necessity of the Ara-box the last two bases of the Ara-box were exchanged in pSVAaraFX-LacS-xylR, creating pSVAaraBox1 (Figure 3D) and pSVAaraBox2 (Figure 3E), respectively. These single point mutations in the Ara-box nearly abolished all promoter activity (Figure 3C) and therefore show the importance of the conserved motif.

### Promotor Activity Assay on Different Carbon Sources

Although a lot of progress is made in establishing a genetic toolbox for *S. acidocaldarius* a high-throughput expression system is lacking. To optimize high-throughput protein expression different plasmids were constructed that provide easy expression of proteins with different promotors and tags (Table 1, detailed vector map shown in Supplementary Figure S4). This toolbox is based on the FX-cloning (Geertsma and Dutzler, 2011). The new expression system allows to insert any sequence of interest into different expression vectors with a variety of N- and C-terminal tags, which have proven to work well in *S. acidocaldarius*.

**TABLE 1:** Summary of all available FX cloning vectors.

Tag	Saci_2122 (ara) promoter	Saci_1165 (mal) promoter	Saci_1938 (xyl) promoter
N-terminal HA	pSVAaraFX-NtHA	pSVAmalFX-NtHA	pSVAxylFX-NtHA
N-terminal 6xHis	pSVAaraFX-NtH6	pSVAmalFX-NtH6	pSVAxylFX-NtH6
N-terminal StrepII	pSVAaraFX-NtS	pSVAmalFX-NtS	pSVAxylFX-NtS
N-terminal Twin-Strep	pBSaraFX-NtSS	–	–
N-terminal 10xHis + StrepII	pSVAaraFX-NtH10S	pSVAmalFX-NtH10S	pSVAxylFX-NtH10S
C-terminal HA	pSVAaraFX-HA	pSVAmalFX-HA	pSVAxylFX-HA
C-terminal 6xHis	pSVAaraFX-H6	pSVAmalFX-H6	pSVAxylFX-H6
		–	–
C-terminal StrepII	pSVAaraFX-S	pSVAmalFX-S	pSVAxylFX-S
C-terminal Twin-Strep	pBSaraFX-CtSS	–	–
C-terminal StrepII + 10xHis	pSVAaraFX-SH10	pSVAmalFX-SH10	pSVAxylFX-SH10
No tag	pSVAaraFX-Stop	pSVAmalFX-Stop	pSVAxylFX-Stop

In difference to the established and general available FX-cloning system at Addgene repository, the suicide selection cassette was replaced by a blue/white selection cassette based on the *E. coli lacZ* gene. The gene of interest can be amplified using the primers designed on fxcloning.org and cloned into the target plasmid by simultaneous *Sap*I restriction, followed by ligation and transformation (see Supplementary Figure S3).

**FIGURE 4:**
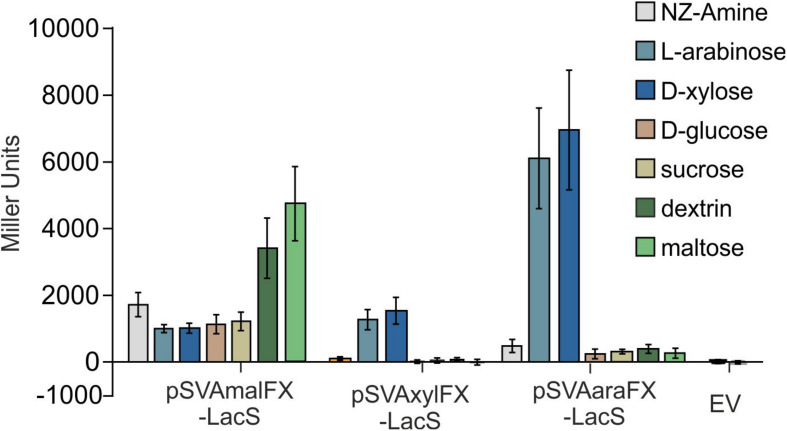
β-galactosidase activity of MW001 transformed with pSVAmalFX-LacS, pSVAxylFX-LacS and pSVAaraFX-LacS grown on different sugars. The strains were cultured on 0.1% N-Z-Amine (gray) and supplemented with 0.2% L-arabinose (light blue), 0.2% D-xylose (dark blue), 0.2% D-glucose (orange), 0.2% sucrose (yellow), 0.2% dextrin (dark green) or 0.2% maltose (light green). The empty vector (EV) was grown on NZ-Amine and NZ-Amine with 0.2% L-arabinose. Activity is shown in Miller Units.

Like other expression systems for *Sulfolobales* (Albers et al., 2006; Peng et al., 2012), these expression plasmids can be induced by addition of different carbohydrates. The system provides two different pentose-inducible promotors (P_xyl_/P_saci___1938_) and (P_ara_/P_saci___2122_) both inducible by D-xylose, L-arabinose, which serve as carbon sources for *S. acidocaldarius* and D-arabinose as artificial inducer and a third promoter, which is inducible with either maltose and dextrin (P_mal_/P_saci___1165_). In addition, the system allows for expression with different C-terminal and N-termial tags (Table 1) and is suitable for both cloning via the class IIS restriction enzyme *Sap*I, or standard cloning with *Nco*I in combination with another restriction enzyme of the MCS. To compare the promoter activities on different carbon sources LacS was cloned behind all three different promotors and measured in an beta-galactosidase assay (Figure 4). The plasmids with P_mal_ show highest β-galactosidase activity upon maltose and dextrin induction, whereas the P_xyl_ and P_ara_ plasmids show induced activity upon induction with the pentoses D-xylose and L-arabinose. Both show relatively low basal activities for other sugars.

**FIGURE 5:**
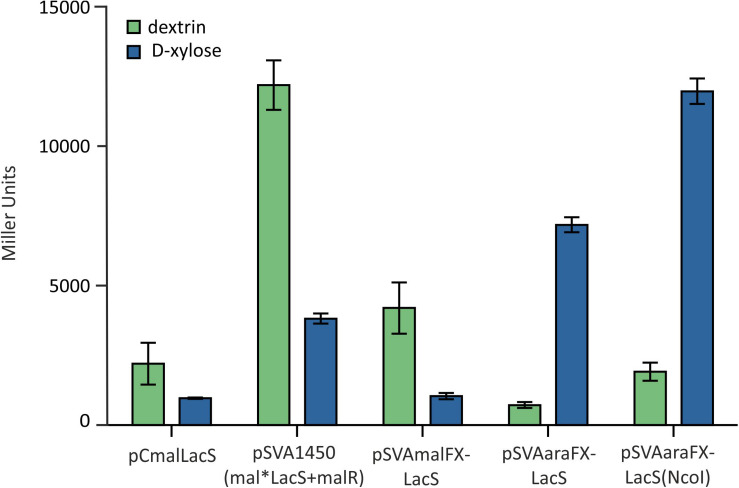
β-galactosidase activity of MW001 transformed with the indicated different expression vectors. The strains were cultured on 0.1% N-Z-Amine and supplemented with 0.3% dextrin (green) or 0.2% D-xylose (blue).

Notably, by using the *Sap*I cloning strategy for simultaneous restriction of the insert and the backbone vector in *S. acidocaldarius*, two additional amino acids alanine and serine are inserted behind the start methionine in the expressed proteins. To analyze for the influence of this modification at the translation start, LacS was cloned again into pSVAaraFX-Stop via classic double restriction using *Nco*I and *Xho*I, avoiding the addition of the two extra amino acids. In the subsequent ONPG assay the different vector constructs were compared and the vector pSVAaraFX-LacS(*Nco*I) showed a significant higher expression than pSVAaraFX-LacS (*Sap*I cloning strategy) (Figure 5), indicating that the additional two amino acids interfere with transcription/translation and thus reduce expression of the *lacS* gene. However, for other genes this effect has not been observed (data not shown). Therefore, it has to be tested for each target gene whether this causes a problem or not. As shown in Figure 5 the more than 3.5kb smaller pSVAaraFX-LacS(*Nco*I) reaches the same expression level for *lacS* like the maltose inducible pSVA1450, which carries an extra copy of the regulator *malR*.

In order to confirm the functionality of the system we cloned the previously described esterase *saci_1116* (Zweerink et al., 2017) via the *Nco*I and *Xho*I restriction site in pSVAaraFX-SH10 and pSVAaraFX-NtH10S with C-terminal Strep-tagII-10xHis-tag and N-terminal 10xHis-Strep-tagII, respectively. Cells were grown in Brock medium supplemented with 0.1% N-Z-Amine and dextrin or D-xylose, respectively (Supplementary Figure S5). For *saci_1116* expression the C-terminal tag showed higher expression. In addition, only in D-xylose induced cells the expression was significantly enhanced whereas in presence of dextrin almost no expression was observed. This demonstrates again that the pentose-inducible vectors show only very low basal expression.

## Discussion

In this study, we identified a positive regulator responsible for controlling the expression of the genes involved in pentose import as well as pentose degradation in *S. acidocaldarius*: XylR. In *S. acidocaldarius*, *xylR* (*saci_2116*) can be found in the genetic neighborhood of the ABC-transporter genes (*saci_2120*–*2122*) responsible for pentose (i.e., D-xylose and L-arabinose) uptake. Based on the data presented in this paper, we propose that XylR is an activator that binds the Ara-box in the promotor region of genes involved in pentose uptake and degradation. In the absence of XylR, *S. acidocaldarius* shows significant growth retardation when grown on pentoses, and qRT-PCR studies showed significantly less transcription of the genes involved in uptake of pentose sugars [XylF, SBP encoding gene (*saci_2122*)]. However, transcription of *xylR* itself does not seem to be regulated at transcriptional levels, suggesting the existence of a different mechanism of *xylR* activation, e.g., via posttranslational modification or further alternative TFs acting at a higher hierarchical level.

The Ara-box motif AACATGTT was first described in *S. solfataricus* in the upstream region of D-arabinose-induced genes (Brouns et al., 2006). Later, the motif was found to be conserved among different *Sulfolobales* species and shown to be essential for the promotor activity (Peng et al., 2009, 2011, 2012; Wagner et al., 2018) and successfully used in early *Sulfolobus* expression systems (Albers et al., 2006). Mutagenesis of the Ara-box in combination with the LacS reporter gene assay confirmed that the conserved Ara sequence motif as well as XylR is required for L-arabinose induced expression in *S. acidocaldarius*. Using growth studies as well as the *lacS* reporter gene assay we demonstrate that the *S. islandicus* XylR homolog could indeed complement the *S. acidocaldarius* deletion mutant. Therefore, we postulate that we have identified XylR (saci_2116) as transcriptional activator of the pentose regulon in Sulfolobales. It is also interesting to note, that the regulatory elements have been conserved among the Sulfolobales, although *S. acidocaldarius* was shown to have acquired the L-arabinose/D-xylose ABC transporter from bacteria (Wagner et al., 2018).

To further improve the *S. acidocaldarius* expression system, we combined the previously established vector system with the newly identified pentose inducible promoters as well as FX cloning and constructed a whole cassette of different vectors from which genes can be expressed in *S. acidocaldarius*. Next to the standard His- and Strep-tags for immunodetection and simple purification in any scale, a HA-tag was chosen because of its high sensitivity and selectivity with very low background in *S. acidocaldarius* cell extracts. Importantly, we could show that in contrast to the so far used maltose inducible promoter, the pentose (D-xylose/L-arabinose) inducible promoters are less leaky in conditions where the inducing sugars are not added, therefore allowing for the expression of toxic gene products. Further on, since *S. acidocaldarius* is not able to utilize D-arabinose as carbon source, D-arabinose can be used as steady inducer similar to isopropyl-β-D-thiogalactopyranosid (IPTG). This is especially interesting for mutational analysis/complementation studies of genes involved in sugar metabolism as well as expression studies e.g., overexpression of regulatory proteins, where the induction of protein expression should not induce the utilization of alternative carbon sources and thus changes in the central carbohydrate metabolism of *S. acidocaldarius*. This new tool kit will further help to establish *S. acidocaldarius* as a model organism for research in crenarchaea and its use biotechnology (Quehenberger et al., 2017).

## Data Availability Statement

All datasets presented in this study are included in the article/Supplementary Material.

## Author Contributions

NK, MW, and BW executed all experiments related to XylR. AW planned and cloned the FX cloning cassette vectors and performed all test involving these. S-VA and BS conceived, supervised, and analyzed the experiments. All authors wrote the manuscript.

## Conflict of Interest

The authors declare that the research was conducted in the absence of any commercial or financial relationships that could be construed as a potential conflict of interest.
