# Supplementary material for: Identification of XylR, the Activator of Arabinose/Xylose Inducible Regulon in Sulfolobus acidocaldarius and Its Application for Homologous Protein Expression
Source: Front Microbiol. 2020 May 26;11:1066. doi: 10.3389/fmicb.2020.01066 (PMC7264815; doi:10.3389/fmicb.2020.01066)
Supplement: Supplementary file 1 [file Data_Sheet_1.pdf]

Supplement to

Identification of XylR, the activator of arabinose/xylose inducible regulon in *Sulfolobus acidocaldarius* and its application for homologous protein expression

Nienke van der Kolk[1], Alexander Wagner[1,2], Michaela Wagner[1,3], Bianca Waßmer[1], Bettina Siebers[2], Sonja-Verena Albers[1,#]

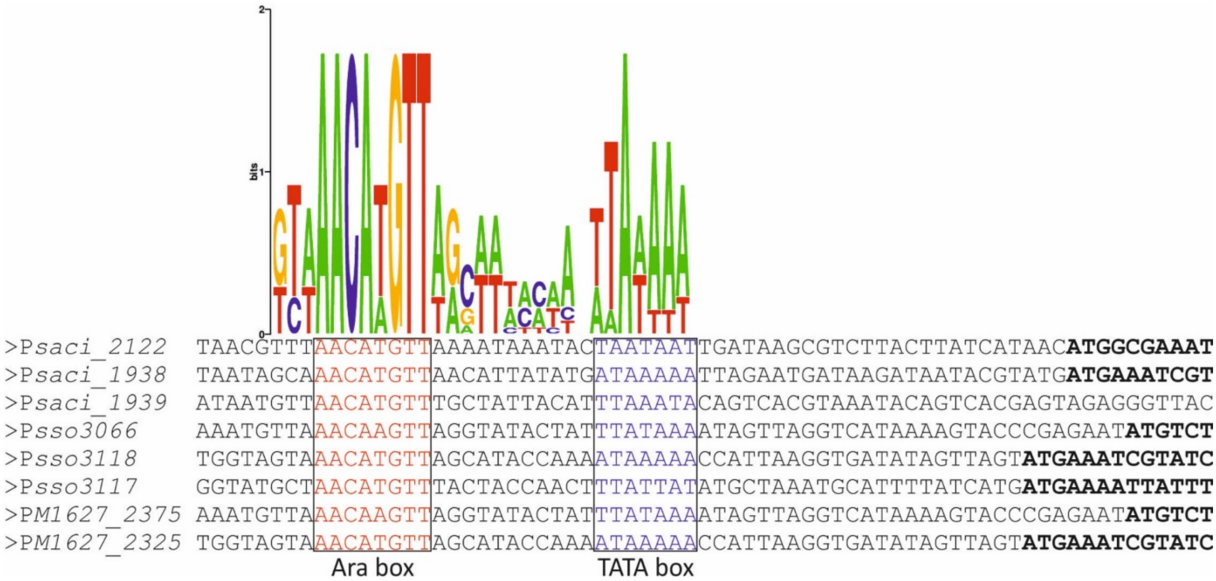

Figure S1. Alignment of the promoters of the genes which are regulated by XylR in *S. acidocaldarius* (*saci_2122*, *saci_1938* and *saci_1939*) and the ones containing the Ara-box from *S. solfataricus* (*sso3066, sso 3118 and sso3117*) and *S. islandicus* (*m1627_2375* and *m1627_2325*).

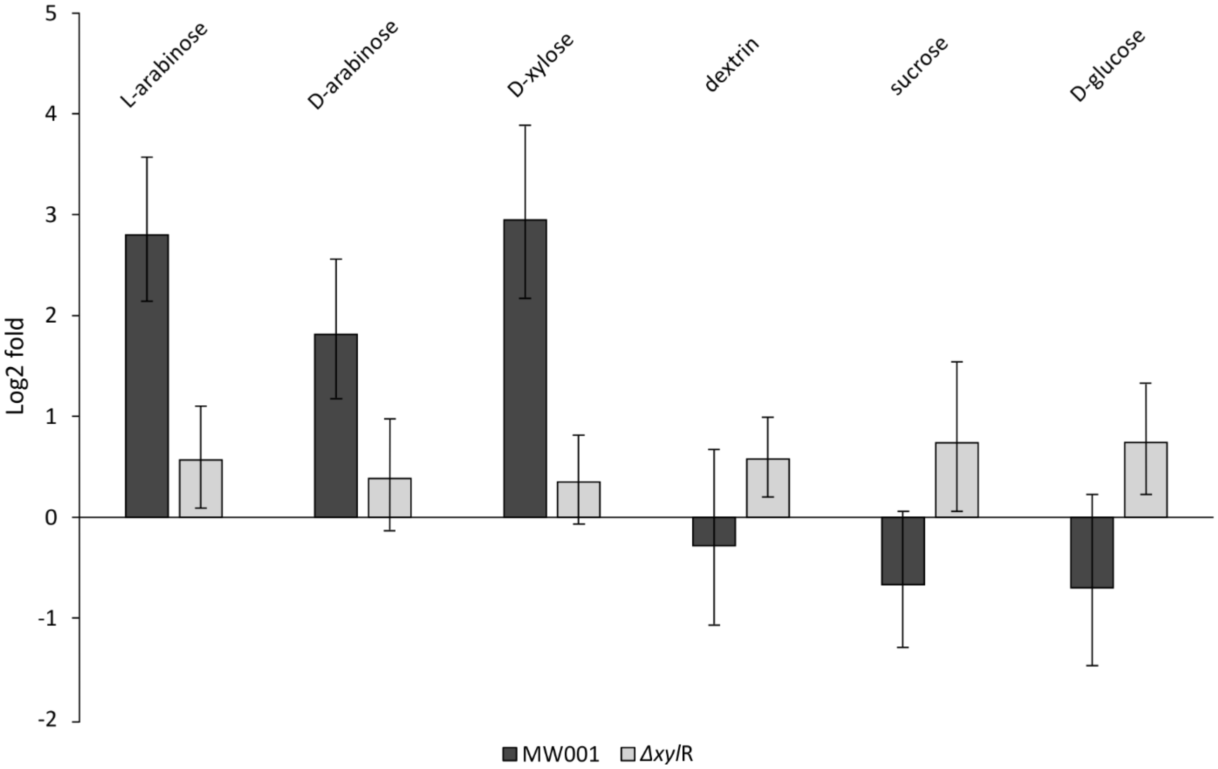

Figure S2. Transciption levels of the gene encoding KDXD/KDAD (*saci_1939*) in the parental strain MW001(dark grey) and ΔxylR deletion mutant (light grey) when grown on different sugars. The strains were cultured on 0.1% N-Z-Amine and supplemented with 0.2% L-arabinose, 0.2% D-arabinose, 0.2% D-xylose, 0.2% dextrin, 0.2% sucrose or 0.2% D-glucose. Bars indicate the sugar-specific transcription compared to cells only grown on N-Z-Amine on log_2-fold

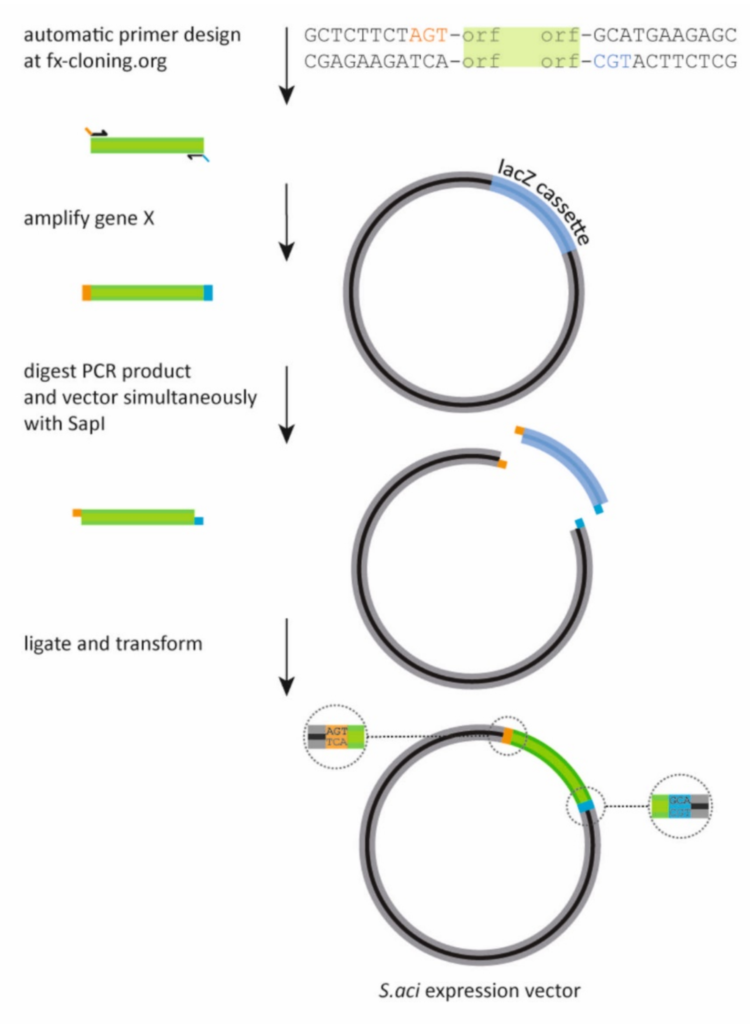

Figure S3. Scheme of the FX cloning method. Adapted from [18]

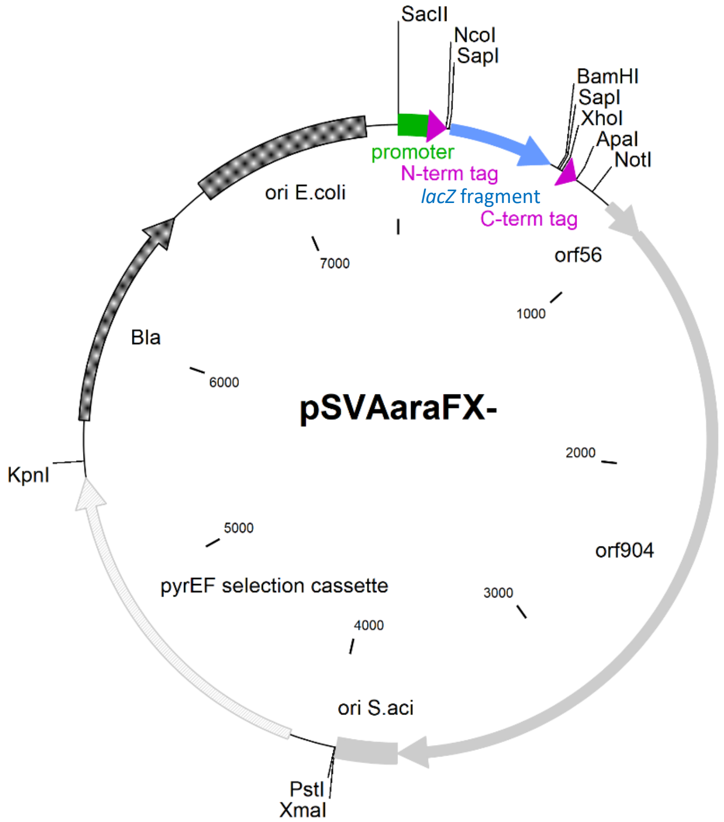

Light grey: *S.aci* plasmid replication elements
Dark grey: *E.coli* replication elements
Green: promoters
Blue: cloning cassette with lacZ fragment
Pink: N- or C-term tags

Saci_2122 (ara)

***SacII* CCGCGG**TATAAATACTAACTGCAATATTATATCTATAATTATTACTCAGCGTTTATAACGTTTAACATGTTAAAATAAATACTAATAATTGATAAGCGTCTTACTTATCATA

Saci_1938 (xyl)

SacII
CCGCGGTTATTGTAACCCTCTACTCGTGACTGTATTTACGTGACTGTATTTAAATGTAATAGCAAACATGTTAACATTATATGATAAAAATTAGAATGATAAGATAATACGTA

Saci_1165 (mal)

***SacII* CCGCGG**CTAATTAATAATACTAATATCATTTCGTAAACTACATCTTATAATACTTAAGTTGACATGTTCAACGGAGGTGTCCTTAAGTTTAGACCTTAAATATTTTA TATATATATTAAGTTTATAAATAATTACGTGATTAAGTTAA

NcoI **SapI** **AatI** **NheI** **SalI** **BamHI** **SapI** **XhoI**

CCATGG CTAGTTG **AAGAGC** … lacZ fragment 416 bp … **GACGTC GCTAGC GTCGAC GGATCC** G **GCTCTTC** AGCA **CTCGAG** TAA

ApaI **Terminator region** **NotI**

GGGCCC ACTTTCTCAAGTCTCACTATACCAAATGAGTTTTCTTTTAATCTTATTCTAATCTCATTTTCATTAGATT **GCGGCC**

NtS

AC**ATG**GCGTGGAGTCATCCACAATTTGAGAAGG

NtHA

AC**ATG**GCGTACCCGTATGACGTTCCGGACTACGCGG

NtH10S

AC**ATG**GCGCATCACCATCATCACCATCATCACCATCATTCCGGATGGAGTC ATCCACAATTTGAGAAGG

NtH6

AC**ATG**GCGCATCACCATCATCACCATG

NtSS

AC**ATG**GCGTGGAGTCATCCACAATTTGAGAAGGGTGGAGGTTCCGGAGG TGGATCGGGAGGTTCTGCATGGTCACATCCTCAATTCGAAAAGGGAGGTT

SH10

TGGAGTCATCCACAATTTGAGAAGCATCACCATCATCACCATCATCACCAT CATTGA

S

TGGAGTCATCCACAATTTGAGAAGTAA

HA

TACCCGTATGACGTTCCGGACTACGCGTAA

H6

CATCACCATCATCACCATTAA

CtSS

AGCGCTTGGTCACATCCTCAATTTGAGAAAGGTGGAGGTTCCGGAGGTG GATCGGGAGGTTCTGCATGGAGTCACCCACAGTTCGAAAAGTAA

Fig S4. Detailed vector map of all FX cloning vectors. The vector map is shown and in the inserts the sequences of the different tags dependend on which vector is described.

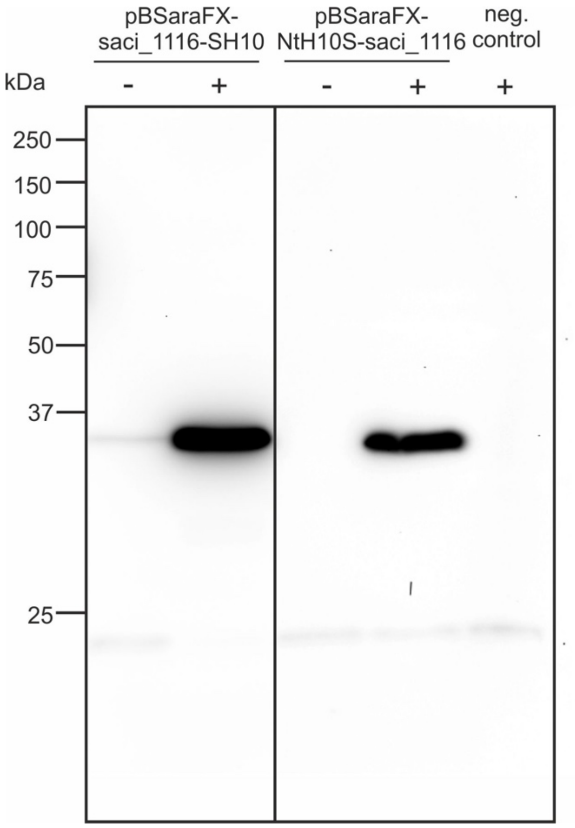

Figure S5. Immunodetection of the esterase Saci_1116 after cloning in pBSaraFX-SH10 and pBSaraFX-NtH10S and expression in *S. acidocaldarius*. Cells were grown in Brock medium supplemented with 0.1% N-Z-Amine and 0.3% dextrin (-) or D-xylose (+). Similar amounts of whole cells were boiled for 5min in SDS loading buffer and separated via SDS-PAGE. Western blot transfer occurred on PVDF membrane using the Trans-Blot Turbo machine from Bio-Rad. For immunodetection Anti His-tag antibody (Abcam ab184607) was used in 1:10000 dilution.

Supplementary tables

Table S1 strains

**Strain**	**Background strain**	**Genotype**	**Reference**
MW001	*S. acidocaldarius* DSM639	Δ*pyrE* (Δ91-412)	(Wagner et al., 2012)
MW413	*S. acidocaldarius* MW001	Δ*xylR (Δsaci_2116)*	This study
